# The Role in Teledermoscopy of an Inexpensive and Easy-to-Use Smartphone Device for the Classification of Three Types of Skin Lesions Using Convolutional Neural Networks

**DOI:** 10.3390/diagnostics11030451

**Published:** 2021-03-05

**Authors:** Federica Veronese, Francesco Branciforti, Elisa Zavattaro, Vanessa Tarantino, Valentina Romano, Kristen M. Meiburger, Massimo Salvi, Silvia Seoni, Paola Savoia

**Affiliations:** 1 AOU Maggiore della Carità, C.so Mazzini 18, 28100 Novara, Italy; 2 Biolab, PolitoBIOmedLab, Department of Electronics and Telecommunications, Politecnico di Torino, 10129 Torino, Italy; francesco.branciforti@polito.it (F.B.); kristen.meiburger@polito.it (K.M.M.); massimo.salvi@polito.it (M.S.); silvia.seoni@polito.it (S.S.); 3 Department of Translational Medicine, University of Eastern Piedmont, Via Solaroli 17, 28100 Novara, Italy; 4 Department of Health Science, University of Eastern Piedmont, Via Solaroli 17, 28100 Novara, Italy; vanessa.tarantino30@gmail.com (V.T.); 20006900@studenti.uniupo.it (V.R.); paola.savoia@med.uniupo.it (P.S.)

**Keywords:** telemedicine, teledermoscopy, convolutional neural networks

## Abstract

Background. The use of teledermatology has spread over the last years, especially during the recent SARS-Cov-2 pandemic. Teledermoscopy, an extension of teledermatology, consists of consulting dermoscopic images, also transmitted through smartphones, to remotely diagnose skin tumors or other dermatological diseases. The purpose of this work was to verify the diagnostic validity of images acquired with an inexpensive smartphone microscope (Nurugo^TM^), employing convolutional neural networks (CNN) to classify malignant melanoma (MM), melanocytic nevus (MN), and seborrheic keratosis (SK). Methods. The CNN, trained with 600 dermatoscopic images from the ISIC (International Skin Imaging Collaboration) archive, was tested on three test sets: ISIC images, images acquired with the Nurugo^TM^, and images acquired with a conventional dermatoscope. Results. The results obtained, although with some limitations due to the smartphone device and small data set, were encouraging, showing comparable results to the clinical dermatoscope and up to 80% accuracy (out of 10 images, two were misclassified) using the Nurugo^TM^ demonstrating how an amateur device can be used with reasonable levels of diagnostic accuracy. Conclusion. Considering the low cost and the ease of use, the Nurugo^TM^ device could be a useful tool for general practitioners (GPs) to perform the first triage of skin lesions, aiding the selection of lesions that require a face-to-face consultation with dermatologists.

## 1. Introduction

The term telemedicine derives from the Greek word tele meaning distant. The application of telemedicine to dermatology is known as teledermatology (TD), which can be classified into real-time teledermatology (VTC) and store-and-forward teledermatology (SAF) [1].

VTC consists of a live video consultation with the patient, whereas SAF consists of image transmission from the patient to the teleconsultant as the first step, then is followed by a plan of action about diagnosis or management from the consultant. Sometimes, TD can be a hybrid and combine elements of real-time and store-and-forward TD; moreover, TD can use mobile phones and so is defined as mobile-teledermatology [1].

An extension of TD includes teledermoscopy (TDSC), in which doctors consult dermoscopic images transmitted electronically. With dermoscopic patterns being well established, especially for skin malignancies, the combination of TD with TDSC has shown to get better effectiveness than only TD consultations. Indeed, TDSC has been found acceptable and effective in triage and the early detection of skin cancers [1].

Smartphone-based TDSC has improved the quality of capture, storage, and transmission of clinical images. The literature in this field has grown steadily over the past 20 years thanks to the development of a large number of dermatology-related mobile applications [1,2] and portable dermatoscopes that can be connected to smartphones [3,4,5]. TD and TDSC using smartphones are useful not only for patients but also for dermatologists to collectively discuss complex cases by social media platforms [1].

The usefulness of TD has also been highlighted by several studies during the recent SARS-Cov-2 pandemic. Thanks to it, patients suffering from chronic diseases or patients who present the appearance or modification of skin lesions have been able to continue to be assisted, even if remotely [6,7,8].

Recently, many studies have shown high levels of concordance in the diagnosis and management plan between TD and face-to-face (FTF) consultation [9]. For skin cancer, the diagnostic accuracy of FTF consultation remains higher when compared with TD [10]. However, a small-scale, randomized controlled trial comparing all TD modalities and FTF examination found an 85% and 78% concordance in diagnosis and treatment recommended, respectively [2,11].

This work was integrated into the TDSC context since the usefulness of a smartphone device in the acquisition of images of melanocytic and non-melanocytic skin lesions was evaluated.

Over the last few years, the implementation of deep learning and convolutional neural networks (CNNs) for medical image classification has grown exponentially [12]. Recent studies have also shown high levels of accuracy for the classification of skin lesions, including tumors, using CNNs [12].

In this work, we analyzed a smartphone microscope device (Nurugo^TM^ Derma), equipped with a special app, developed by the South Korean company Nurugo^TM^, and able to provide high-resolution images of skin lesions. The images acquired by the Nurugo^TM^ Derma were classified by employing a convolutional neural network (CNN) for the distinction between malignant melanoma (MM), melanocytic nevus (MN), and seborrheic keratosis (SK). To understand the reliability of this device, conventional dermoscopic images of the same lesions were also classified using the same CNN.

## 2. Materials and Methods

### 2.1. Devices’ and Images’ Acquisition

**Figure 1:**
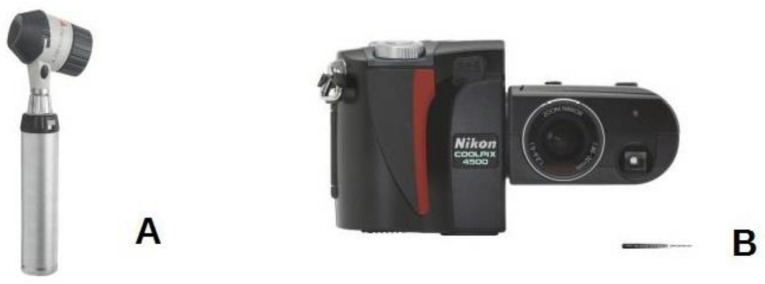
(**A**) Contact dermatoscope HEINE Delta 20T; (**B**) Professional reflex camera NIKON E4500.

For the acquisition of dermoscopic images, a contact dermatoscope HEINE Delta 20T (Figure 1A) was connected to a professional reflex camera (NIKON E4500, Figure 1B) with a photo adapter (SLR HEINE).

**Figure 2:**
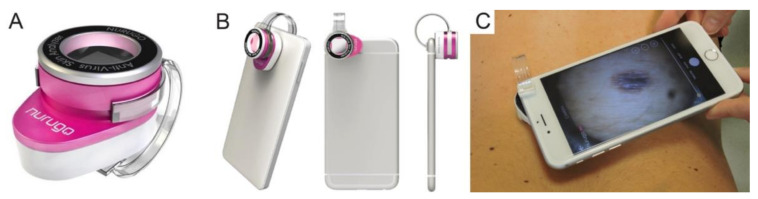
(**A**) Nurugo TM Derma; (**B**) Nurugo TM Derma attached to the smartphone; (**C**) Example of smartphone microscope applied to skin.

For the acquisition of smartphone images, the Nurugo^TM^ Derma was used, an amateur device consisting of a lens that allows skin examination at a microscopic level by conveying the light emitted by the smartphone flash through a system of reflecting prisms (Figure 2A). This device employs a specific app for iOS and Android called “Nurugo Box” and is compatible with most smartphones on the market (in our specific case, iPhones 6, 6s, and 7). It is attached to the smartphone through a plastic clip, correctly aligning the camera and flash (Figure 2B,C).

**Figure 3:**
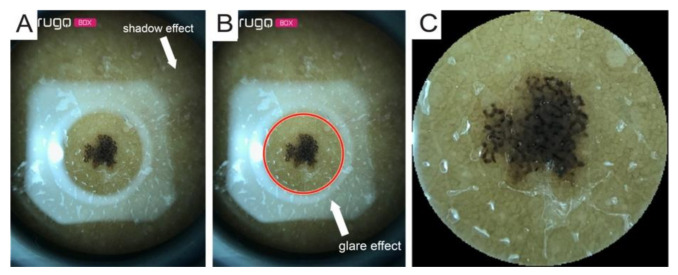
(**A**) Example of original image; (**B**) Example of original image with Hough transform result; (**C**) Cropped image.

Since the device was designed for amateur use, it has some limitations:

(1) Shadow effect: due to not being able to compress the lesions directly using the Nurugo microscope (Figure 3A);
(2) Glare effect: due to the light of the smartphone flash reflected off of the skin (Figure 3B);
(3) The inability to acquire epiluminescence images (the flash of common smartphones does not produce polarized light and, therefore, does not allow the visualization of the structures under the epidermis); and
(4) Impossibility of applying immersion oil to cancel the reflection of light (the device has no support downstream of the lens).

In this study, the last two limitations were overcome using a transparent laboratory slide placed between the smartphone microscope and the skin in order to apply a liquid interface on the skin.

In this way, the images showed the underlying skin structures but remained heavily burdened by the glare effect, which reduced the field of view (FOV), limiting it to a circular area of 3 mm^2^. Therefore, from a single acquisition, it was possible to obtain only a small portion of the lesion. For large lesions, more images were acquired, moving the position of the microscope on the lesion.

### 2.2. Processing

All the images acquired by the dermatoscope had a central circular area with the lesion inside and a large area of black pixels; the images were cropped and rescaled to have the same FOV as the smartphone microscope images. A segmentation algorithm based on the circular Hough transform [13] was developed for the epiluminescence images acquired with the smartphone microscope burdened by the glare effect in order to circumscribe the portion not contaminated by the reflection artifacts (Figure 3).

The dermoscopic and Nurugo^TM^ images were acquired at Dermatologic Clinic (Maggiore della Carità Hospital, University of Eastern Piedmont, Novara) and the image analysis was carried out by engineers of the Polytechnic of Turin (Biolab, Polito^BIO^Med Lab, Department of Electronics and Telecommunications).

**Figure 4:**
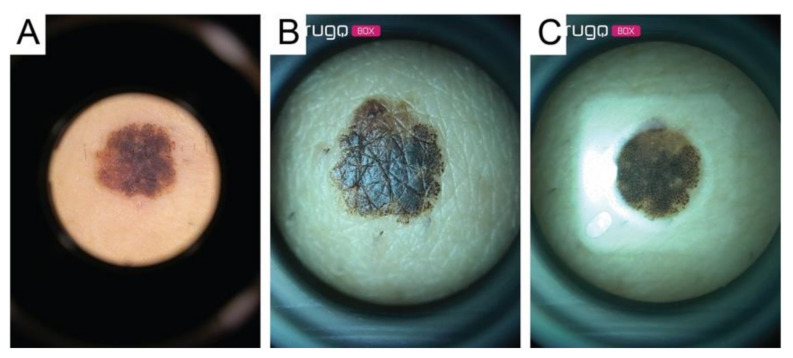
Image of a malignant melanoma acquired through: (**A**) contact dermatoscope; (**B**) Nurugo ^TM^ microscope without laboratory glass; (**C**) Nurugo ^TM^ microscope with laboratory glass. The arrows in Figure 4A,B clearly point out where the shadow effect and glare effect can be easily seen.

**Figure 5:**
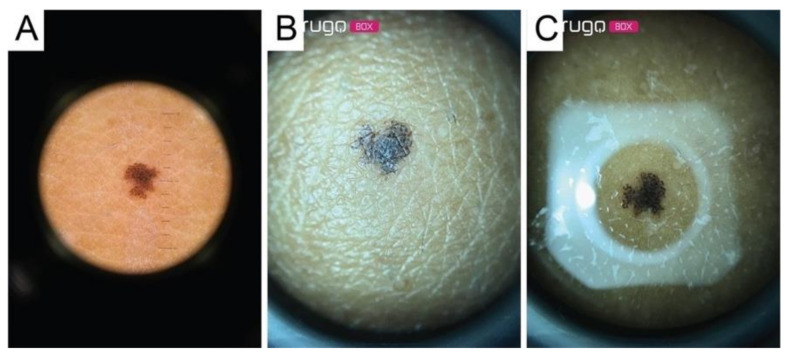
Image of a melanocytic nevus acquired through: (**A**) contact dermatoscope; (**B**) Nurugo ^TM^ microscope without laboratory glass; (**C**) Nurugo ^TM^ microscope with laboratory glass.

**Figure 6:**
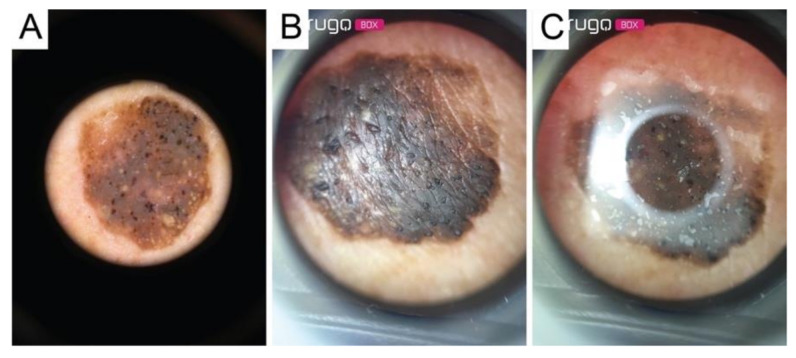
Image of a seborrheic keratosis acquired through: (**A**) contact dermatoscope; (**B**) Nurugo ^TM^ microscope without laboratory glass; (**C**) Nurugo ^TM^ microscope with laboratory glass.

The data set analyzed included 18 images of malignant melanomas (MM), 39 melanocytic nevi (MN), and 21 seborrheic keratoses (SK). All lesions were acquired both with the conventional contact dermatoscope and with the Nurugo^TM^ microscope, with and without laboratory glass slide (Figure 4, Figure 5 and Figure 6).

The image acquisition was done in the surgery room before excision for malignant melanoma and atypical nevi, and during routine dermatological visits for benign lesions. The images were encoded to maintain the anonymity of the patients (only adults), who signed the relative informed consent to participate in the study. Each lesion was evaluated by three different expert dermatologists and classified based on visual clinical and dermoscopic parameters. For malignant melanoma and atypical nevi, the definitive diagnosis was obtained by histological examination. For benign lesions, it was determined according to dermoscopic parameters. The present study was conducted according to the Declaration of Helsinki and it was approved by the Local Ethical Committee on 12 December 2018 (protocol CE 173/18; Acronym Teledermatology).

### 2.3. CNN Classification Algorithm

There are various pretrained models of CNNs in the literature, which can be employed for transfer learning, which is useful when only a small database is available.

**Figure 7:**
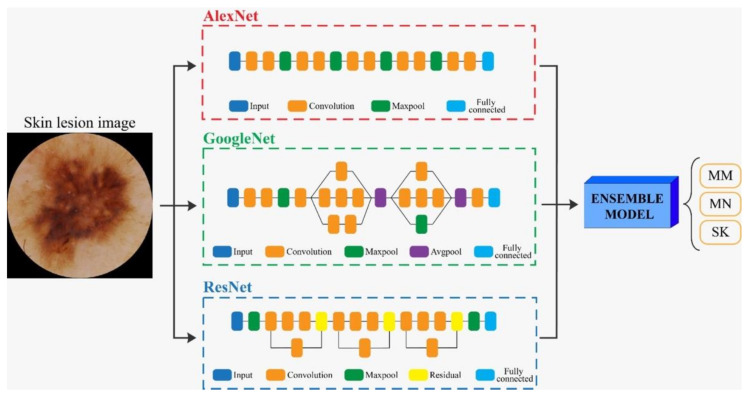
Flowchart for the classification of skin lesion images. Three different CNNs’ (AlexNet, GoogleNet, and ResNet) were employed for classification. Then, an ensemble model averaged all the CNNs’ predictions to obtain the final label of the image. MM: malignant melanoma, MN: melanocytic nevus, SK: seborrheic keratosis.

The creation of CNN involves several stages (Figure 7):

(1) Training phase in which networks learn from the examples provided (training-set images for learning and validation-set images to test the learning level).
(2) Evaluation of the final performances on the test set images to understand the model’s ability to classify new images, not used during training.
(3) In our study, transfer learning was applied using three different CNN architectures: *AlexNet*, *GoogleNet, and ResNet* [14]. The *AlexNet* [15] employed a series of convolutional layers to extract a higher-level representation of the image content. The *GoogleNet* [16] was organized to concatenate convolutional layers having different kernel sizes. The *ResNet* [17] adopted skip connections and batch normalization to perform the classification task.
(4) Finally, we created an ensemble model that combined the predictions of the three deep networks (AlexNet, GoogleNet, and ResNet). Specifically, the probability of the ensemble model was obtained as the average of the three output probabilities from each single CNN. Then, the final, predicted label was equal to the predicted label with maximum probability over all classes (MM, MN, SK).

All these CNNs were trained on an open-source collection of dermatological images, the ISIC (International Skin Imaging Collaboration) archive [18]. A sub-data set from the entire database was employed (MSK ISIC), using only MM, MN, and SK images (Table 1) that presented resolution and dimensions similar to those acquired by the smartphone microscope and cropped with the FOV radius of 3 mm. This significantly reduced the size of the training set, as only images with a visual ruler on the image were able to be included.

Finally, the CNNs were tested on three different test sets, as shown in Table 1, to evaluate the performance variations between the different methodologies, identifying the most reliable in the recognition of the different types of images generated by the different devices. In particular, the images acquired at the Dermatologic Clinic were used in the CNN testing phase, to verify the diagnostic validity of the images of the same lesions acquired with Nurugo^TM^ Derma compared to those acquired with the clinical dermatoscope.

To validate the classification, the following parameters were evaluated: (1) accuracy; (2) sensitivity and specificity; (3) positive (PV+) and negative (PV−) predictive value; and (4) F1 score (measure of total model accuracy by combining precision and recall), where the MM images were considered as true positives and the MN and SK images were considered as true negatives.

Furthermore, a receiver operating curve (ROC) analysis was done and the area under the curve (AUC) was computed for each classification method.

**Figure 8:**
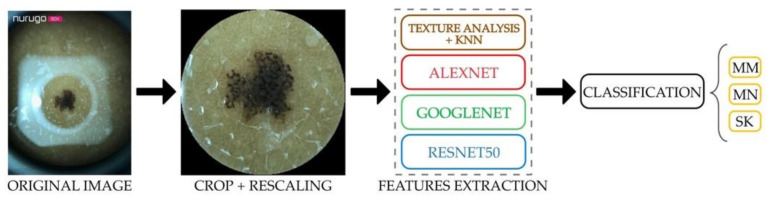
Block diagram of proposed approach.

In Figure 8 a block diagram of the proposed approach is shown.

## 3. Results

For each test set, we computed the performance of: (1) three expert dermatologists (D1, D2, D2); (2) a machine learning algorithm [19] based on traditional texture analysis [20]; (3) three deep neural networks (AlexNet, GoogleNet, ResNet), and (4) ensemble model.

### 3.1. Performance on ISIC Images (Test Set 1)

From the performance analysis (Table 2), it emerged that the ensemble model methodology guarantees the best results overall.

To quantify the reliability of the methods previously described, a comparison was subsequently made between the performances achieved by texture analysis combined with a K-Nearest Neighbor (KNN) classifier, individual CNN architectures, the ensemble model, and those obtained by three different dermatologists on the same data set comprised of the ISIC images (i.e., test set 1).

The comparison between the values of the three CNNs, the ensemble model, and those obtained by the dermatologists demonstrated that the ensemble model approach showed better levels of accuracy, sensitivity, and F1 score than both the individual CNNs, except GoogleNet in terms of sensitivity, and the evaluation of experts while obtaining lower scores regarding specificity.

All CNNs were in line with the experts, and the ensemble model even exceeded their performance, not only in terms of sensitivity, but also in overall accuracy, PV-, and F1 score. It is possible to assert that all the individual CNNs were moderately accurate classifiers, far more advanced than texture analysis with a KNN. In particular, as expected, the ensemble model that combined the predictions of the three deep networks gave forth the best overall performance.

**Figure 9:**
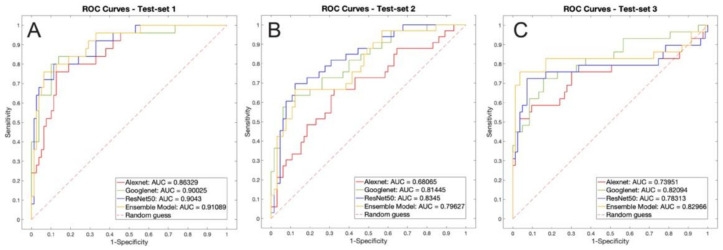
ROC analysis of the three test sets. (**A**) test set 1, ISIC images; (**B**) test set 2, new dermatoscopic images; (**C**) test set 3, Nurugo TM images.

The ROC curve of test set 1 can be seen in Figure 9A.

### 3.2. Performance on Dermatoscope Images (Test Set 2)

To quantify the ability to recognize images acquired with various devices and different from those used for training, the three methodologies were tested using the test set 2, containing images acquired at the Dermatologic Clinic with the contact dermoscope previously described (Table 3).

As expected, since the various methodologies were trained on a limited data set coming from the large ISIC database, the different architectures showed difficulties in recognizing lesions that presented lower quality in terms of pixel/mm resolution. The presence of artifacts such as bubbles and lack of focus contributed to making the classification even more difficult. Similarly to test set 1, the images were manually classified by three different expert dermatologists. As observed in Table 3, the performance of the dermatologists were overall higher compared to all automated methods, including the ensemble model. Among the different CNNs, GoogleNet showed the highest sensitivity, but ResNet proved to be more stable with a higher F1 score than all models and a specificity in line with the experts. Nevertheless, it should also be specified that some dermatoscope images looked familiar to the experts, who had been able to observe the clinical appearance of the lesions before surgical excision, which certainly contributed to bias of the manual results against the CNNs’ performance.

The ROC curve of test set 1 can be seen in Figure 9B.

### 3.3. Performance on Nurugo^TM^ Derma Images (Test Set 3)

Finally, we evaluated the diagnostic utility of the images acquired in epiluminescence with the Nurugo^TM^ Derma, verifying if they could be interpreted as well as dermatoscope images by a CNN.

The images acquired with this device presented, as previously stated, several challenges, such as the presence of numerous air bubbles, the lack of focus if not acquired properly, and the presence of streaks probably attributable to the physical composition of the laboratory slide. Table 4 shows the performances achieved by the different dermatologists and automated algorithms.

It is possible to note how, like test set 1 and test set 2, texture analysis did not provide convincing results. Instead, individual CNNs showed promising performance in terms of specificity, but they failed to rival the experts in terms of accuracy and sensitivity. However, the combination of the predictions of the individual deep networks, implemented by the ensemble model, succeeded in balancing the gaps of the individual CNN. It showed performance in line with the experts in terms of accuracy and sensitivity but scoring far higher in specificity and competing in PV+, PV-, and F1 score.

Despite the lack of focus, the streaks caused by the slide, and the air bubbles caused by the interface fluid, the Ensemble model proved to be a solid and effective classifier on these types of images acquired with a smartphone.

Looking at the performances of the dermatologists, it was possible to notice how, despite having tested the same images, acquired with a different device (Nurugo^TM^ Derma), they were slightly worse than in test set 2 (lower F1 score in all three cases), showing how the artifacts produced by the device represented a limitation factor for both deep learning automated algorithms and clinicians.

The ROC curve of test set 1 can be seen in Figure 9C.

## 4. Discussion

In this study we evaluated the possibility of making accurate diagnoses of melanocytic and non-melanocytic skin lesions on images acquired by the smartphone camera using the Nurugo^TM^ Derma amateur device and on images of the same lesions acquired by a portable dermoscope and a digital camera, using deep neural networks. The purpose was to provide information on the clinical and diagnostic validity of the Nurugo^TM^ Derma device, in the context of TDSC. Indeed, the current health situation, consequent to the COVID-19 pandemic, causes the growing need to acquire images of skin lesions even with amateur and low-cost devices. On the other hand, the quality of the images is essential to be able to discriminate suspicious lesions that need to be subjected to further investigation.

Our results showed that the ensemble model trained on the images of the ISIC database [18] obtained a maximum prediction accuracy of 79.8% and a maximum F1 score of 70%, even exceeding the performances achieved by the three dermatologists who examined the same images (average accuracy of 71% and average F1 score of 55%). As for dermoscopic images, the maximum accuracy was achieved by the ResNet model (70.4%), while the maximum F1 score was 69.6%.

Finally, with the images acquired through Nurugo^TM^ Derma, the ensemble model reached an overall accuracy of 83.9%, a sensitivity of 72.4%, a specificity of 97.3%, and an F1 score of 80.8%. The results obtained are encouraging, demonstrating how also an amateur device can be helpful for clinical analysis, with all the related limits and possibly implementing improvements and measures that can increase its performance.

To the best of our knowledge, to date there are no comparable literature studies that have used similar devices. However, some considerations on Nurugo^TM^ Derma can be made, analyzing some studies of the last decade about TD and TDSC.

Recently, Munoz-Lopez et al. [21] (2021) conducted a prospective, real-life study with the aim of assessing an AI (artificial intelligence) algorithm’s performance, published by Han et al. in 2020 [22], for the diagnosis of skin diseases. Patients submitted photographs of one or more skin conditions acquired using a smartphone prior to or during a TD evaluation. The AI web application, following the upload of the images, output three diagnoses ranked in order of probability. Finally, the algorithm’s performance was compared to those of physicians with different levels of experience. Similarly to our findings, the accuracy of the algorithm’s diagnosis was inferior to the accuracy of dermatologists. Nonetheless, the authors concluded that the use of the AI web application could be a valuable collaborative tool, enhancing the confidence and accuracy of physicians.

The algorithm of Han et al. had already been tested by Navarrete-Dechent in March 2018 [22], submitting to the web application 100 selected images of biopsied cutaneous melanomas, basal cell carcinoma, and squamous cell carcinoma originated from Caucasian patients. Overall, the computer classifier matched histopathological diagnosis only in 29 out of 100 lesions (29%), suggesting that CNN training requires the largest data sets including the full spectrum of human population and clinical presentations.

In 2012, Lamel et al. [23] evaluated the diagnostic concordance between FTF consultations and TD in patients undergoing screening for skin cancers. Clinical images were transmitted through a smartphone, without device integrated into the camera. Digital images of 137 skin lesions were acquired using Google Android G1 (HTC Corporation, Taoyuan, Taiwan), a smartphone with an integrated 3.2-megapixel autofocus camera, equipped with the ClickDerm app (Click Diagnostics, Boston, MA), developed to facilitate the remote diagnosis of skin diseases by dermatologists. During this study, one dermatologist performed the FTF evaluations, while another dermatologist assessed digital images captured by the smartphone, with a diagnostic concordance of 62%. In our study, the diagnostic accuracy of the teledermatologist (average accuracy of D1, D2, D3 = 89%) was higher and comparable to that of the CNN on image recognition of the device under study using the ensemble model (83%). Moreover, the Nurugo^TM^ Derma offers the possibility to acquire images showing the dermoscopic features of the lesions.

A further aim of the study by Börve et al. (2013) [5], the main objective, was to determine the diagnostic accuracy of a mobile TDSC and, subsequently, the diagnostic concordance between TDs and an FTF dermatologist. The study included 62 patients and was conducted using a smartphone (iPhone 4, Apple Inc., Cupertino, CA, USA), a dermoscope connected to the smartphone (FotoFinder Handyscope, FotoFinder Systems GmbH, Bad Birnbach, Germany), a TDSC platform (Tele-Dermis, iDoc24 AB, Gothenburg, Sweden), and a new iPhone app (iDoc24 AB, Gothenburg, Sweden) installed on the smartphone. The diagnosis provided by the FTF dermatologist was correct for 46 lesions (66.7%), showing an accuracy statistically higher than TDs 1 (50.7%) and similar to TDs 2 (60.9%). Based on this study, it was shown that this mobile TDSC solution allows us to achieve diagnostic accuracy comparable to that of an FTF dermatologist.

The aim of the study [24] was also to test this app to evaluate its possible usefulness in the triage of patients with suspicious skin lesions who are referred to dermatologists by general practitioners (GPs). However, the study showed several limitations, because only lesions requiring biopsy or excision were included and the TDs were aware of this, resulting in a possible bias in the assessment. Furthermore, all images were acquired by the FTF dermatologist, who had experience in the use of imaging equipment, while the image quality may be lower if smartphones are used by GPs. On the contrary, Nurugo^TM^ Derma is a very intuitive tool, accessible and easy to use even by nonspecialists, who, following adequate training, could obtain valid images that identify suspicious characteristics. In fact, the three dermatologists obtained comparable accuracy in test 2 (dermoscopic lesions) and test 3 (same lesions but acquired with Nurugo^TM^ Derma). This shows that for a specialist the image of the Nurugo^TM^ is comparable to that of a traditional dermoscope. In addition, the ensemble model also performed well in the classification of Nurugo^TM^ images, reaching an accuracy of 84%. Also, Nurugo^TM^ Derma is a low-cost device (about $50), very intuitive and practical, which could be positively accepted by GPs and integrated into clinical practice. Therefore, we believe that this can be a valid screening tool and that its use can allow the patients’ referral with greater appropriateness, discriminating the degree of urgency. In fact, only patients with suspicious or malignant lesions can be urgently referred to a specialist consultation.

Likewise, a recent Norwegian pilot study (Houwink et al., 2020) [25] tested an app (Askin^®^) for smartphones, that allows clinical and dermatoscopic photographs of various skin lesions to be taken and then sent to the dermatologist. Dermatoscopic images were obtained using a dermoscopy lens (AskinScope^®^), to be fixed to the smartphone camera. In this study, the diagnoses obtained by the dermatologists included not only pigmented skin lesions and benign or malignant tumors but also inflammatory skin conditions (i.e., infections, eczema, or chronic ulcers) and uncertain diagnosis lesions (i.e., lesions in which the clinical diagnosis was not possible and differential diagnoses were suggested). It was estimated that the app reduced the need for specialist assessment by around 70%. Therefore, TD and TDSC can be part of a triage system in which patients with suspicious skin lesions can be referred more quickly and correctly to the specialist.

However, at the moment, Nurugo^TM^ Derma is intended for nonmedical use and, therefore, further studies are needed for its validation and to overcome its limitations.

The biggest current limitation is that to obtain dermoscopic-like images, a laboratory slide must be used. However, this approach involved several limitations on the acquired images in this study:

(1) The FOV of the image was excessively restricted by the artifact caused by the glass.
(2) The bubbles created by the interface liquid interfered with the image interpretation.
(3) The use of the slide itself complicated image acquisition, rendering it more time consuming.

As the results showed, the Nurugo^TM^ Derma could be useful as a tool to perform the first triage of skin lesions through a visual analysis of the acquired images, but the current CNN architectures and performances are limiting and the database must be expanded upon to evaluate changes in accuracy and performance improvements.

Similarly, for each of the numerous nonprofessional apps currently available, the possible limitations need to be explained.

Wolf et al. in 2013 [26] published a review in which four of the most downloaded apps on smartphone platforms were analyzed, for a total of 188 lesions, belonging to one of the following categories: invasive melanoma, melanoma in situ, lentigo, benign nevus, dermatofibroma, seborrheic keratosis, and hemangioma. Of these lesions, 60 were melanomas, while the remaining 128 were benign. The comparison with histology showed a sensitivity ranging from 6.8% to 98.1% and specificity from 30.4% to 93.7%. Therefore, it is also necessary to emphasize the potential dangers of these apps for users who completely rely on them without a critical evaluation. So, users must be aware that the app evaluates the risk that a lesion may be benign or malignant but does not make a diagnosis of certainty. Most of the apps are designed for educational rather than diagnostic purposes and, to date, no method based on an automated algorithm for the analysis of skin lesions shows a sensitivity higher than FTF.

Also, even when used by dermatologists, TDSC has a few limits [27]: The first is the inability to perform a complete full-body examination on patients, with the risk of losing accidentally diagnosed melanomas. If mobile TDSC is used by GPs, there may be a risk of underdiagnosis of clinically significant lesions that are not appreciated by the referring physician. From this is derived the legal risk caused by under- and misdiagnosis. To reduce these complications, a specific training for dermoscopy and use of TDSC devices needs to be adopted, particularly for GPs. On the other side, the present device could be useful for the fast evaluation of a single, and possibly recently appeared, lesion the patient points out, thus allowing to quickly make a clinical decision.

Another limit is, at least in Italy, the regulation from the point of view of reimbursements of this type of service. The development of business models related to TD and TDCS must be taken into consideration as well as the ethical and legal aspects.

In literature, four business models are proposed:

(1) Standard fee-for-service reimbursement from insurance.
(2) Capitated service contracts.
(3) Per-case service contracts.
(4) Direct to consumer [1,28]. In the case of Italy, a fee should be set up to be paid by the patient or by the National Health Service for assisted patients.

## 5. Conclusions

In conclusion, even if with some limitations, the Nurugo^TM^ device could be considered a low-cost and easy-to-use device to perform the first triage of skin lesions, aiding the selection of patients who need a face-to-face consultation by dermatologists.

Also, considering the possibility of reaching patients remotely, also in the event of travel restrictions (such as the recent SARS-Cov-2 pandemic), this method must be strengthened in the future and applied also to the evaluation and monitoring of other skin lesions (e.g., non-melanoma skin cancers or inflammatory cutaneous diseases). Regarding TD and TDSC, our future studies will include increasing the size of the database of images acquired with both a smartphone device and a clinical dermatoscope and the development of low-cost and easy-to-use devices that, after adequate training, can be used also by GPs for the screening of skin lesions that need to be appropriately addressed by a FTF consultation. Moreover, once a larger data set is acquired, we will continue to train and improve the automatic classification network.

## Figures and Tables

**Table 1:** Composition of the training set and the different data test sets employed in this study.

Set Images	MN (Images)	MM (Images)	SK (Images)
Training-set	200	200	200
Test set 1 *	35	25	37
Test set 2 °	39	18	21
Test set 3 §	39	18	21

* Images from MSK ISIC but different from those of the training set. ° Images of skin lesions acquired with a contact dermatoscope. § Images acquired with Nurugo Derma.

**Table 2:** Performances of the three dermatologists (D1, D2, D3) on test set 1 and automated algorithms (texture analysis, AlexNet, GoogleNet, ResNet, ensemble model).

Method	Accuracy	Sensitivity	Specificity	PV+	PV−	F1
D1	68.0%	36.0%	74.7%	51.3%	93.3%	63.5%
D2	70.1%	32.0%	97.0%	80.0%	78.0%	46.0%
D3	75.3%	44.0%	94.0%	73.3%	81.6%	55.0%
Texture analysis	48.5%	44.0%	66.7%	37.9%	72.0%	40.7%
AlexNet	76.0%	80.0%	77.6%	54.1%	92.2%	64.5%
GoogleNet	74.0%	88.0%	76.4%	56.4%	94.8%	68.8%
ResNet	74.0%	80.0%	77.3%	54.1%	92.0%	64.5%
Ensemble model	79.8%	84.0%	81.6%	60.0%	93.9%	70.0%

**Table 3:** Performances of dermatologists and automated methods on test set 2.

Method	Accuracy	Sensitivity	Specificity	PV+	PV−	F1
D1	94.9%	83.3%	79.7%	93.8%	95.2%	88.2%
D2	93.6%	88.9%	78.1%	84.2%	96.6%	86.5%
D3	92.3%	83.3%	79.2%	83.3%	95.0%	83.3%
Texture analysis	31.6%	21.1%	53.3%	25.0%	48.0%	22.9%
AlexNet	56.1%	69.7%	52.5%	44.2%	76.2%	54.1%
GoogleNet	55.1%	81.8%	49.1%	49.1%	81.8%	61.4%
ResNet	70.4%	72.7%	79.0%	66.7%	83.3%	69.6%
Ensemble model	57.1%	75.8%	53.5%	48.1%	79.5%	58.8%

**Table 4:** Performances of dermatologists and automated methods on test set 3.

Method	Accuracy	Sensitivity	Specificity	PV+	PV−	F1
D1	92.3%	77.8%	80.6%	87.5%	93.5%	82.4%
D2	88.5%	66.7%	82.6%	80.0%	90.5%	72.7%
D3	87.2%	72.2%	80.9%	72.2%	91.7%	72.2%
Texture analysis	48.4%	48.3%	50.7%	27.4%	71.7%	35.0%
AlexNet	67.9%	58.6%	85.5%	63.0%	83.1%	60.7%
GoogleNet	70.5%	65.5%	84.5%	63.3%	85.7%	64.4%
ResNet	75.9%	69.0%	90.3%	74.1%	87.8%	71.4%
Ensemble model	83.9%	72.4%	97.3%	91.3%	90.1%	80.8%

## Data Availability

The data used for training and test-set 1 are available at: Available online: https://www.isic-archive.com/#!/topWithHeader/onlyHeaderTop/gallery. The new data presented in this study (test-set 2 and test-set 3) are not publicly available due to privacy issues.
